# Case Report: Rare comorbidity of celiac disease and Evans syndrome: The rare correlation of Celiac and Evans syndrome

**DOI:** 10.12688/f1000research.18182.1

**Published:** 2019-02-14

**Authors:** Syed Mohammad Mazhar Uddin, Aatera Haq, Zara Haq, Uzair Yaqoob

**Affiliations:** 1 Civil Hospital, Karachi, Sindh, Pakistan; 2 Dow University of Health Sciences, Karachi, Pakistan; 3 Jinnah Postgraduate Medical Centre, Karachi, Sindh, Pakistan

**Keywords:** Celiac disease, Evans syndrome, autoimmune disease

## Abstract

**Background: **Celiac disease is an immune-mediated enteropathy due to permanent sensitivity to gluten in genetically predisposed individuals. Evans syndrome is an autoimmune disorder designated with simultaneous or successive development of autoimmune hemolytic anemia and immune thrombocytopenia and/or immune neutropenia in the absence of any cause.

**Case Report: **We report a rare case of Celiac disease and Evans syndrome in a 20-year-old female who presented to us with generalized weakness and shortness of breath. Her examination finding included anemia, jaundice, and raised jugular venous pulse. Her abdominal exam revealed hepatosplenomegaly. Her laboratory values showed microcytic anemia, leukocytosis and thrombocytopenia. To rule out secondary causes of idiopathic thrombocytopenia purpura, we tested viral markers for Human immunodeficiency virus, Epstein bar virus, Cytomegalovirus and performed a *Helicobacter pylori* test, all of which were negative. We also ruled out idiopathic thrombocytopenia purpura associated with any thyroid disorder.  For celiac disease, we took anti-tissue transgulataminase titers of IgA and IgG which confirmed the diagnosis of celiac disease. For the diagnosis of Evans syndrome, despite a negative serum coombs test initially, her bone marrow sample showed a positive Coombs test along with immune mediated hemolytic anemia and immune mediated thrombocytopenia. The patient was treated with prednisone which was tapered off and counseling was provided regarding a gluten free diet.

**Conclusion:** Although rare, tests for Evans syndrome (and other coexisting autoimmune problems) should be performed in patients with celiac disease.

## Introduction

Celiac disease (CD) is defined as an immune-mediated enteropathy due to permanent sensitivity to gluten in genetically predisposed individuals. It is known to affect roughly 1% of the population worldwide. This occurs when genetically predisposed individuals consume gluten, which is a storage protein in wheat, and other wheat related grain species (e.g. barley and rye) ^1^. Several conditions such as dermatitis herpetiformis, autoimmune thyroiditis and type 1 diabetes mellitus has been reported with CD ^2^.

Evans syndrome (ES) is an autoimmune disorder designated with simultaneous or successive development of autoimmune hemolytic anemia (AIHA) and immune thrombocytopenia (ITP) and/or immune neutropenia in the absence of any cause. However, it may be associated with conditions such as systemic lupus erythematosus (SLE), lymphoproliferative disorders or primary immunodeficiencies ^3^. The pathway of the disease is chronic and relapsing, with unknown pathophysiology. Studies have reported that autoantibodies target antigens on red blood cells and platelet, leading to hemolytic anemia and isolated thrombocytopenia ^2^. We report a unique case of 20-year-old female with celiac disease and Evans syndrome (ES).

## Case presentation

A 20-year-old Sindhi female student with no known comorbid presented to the emergency department with a complaint of generalized weakness and shortness of breath over the previous 15 days. According to the patient herself, the generalized weakness was progressive and with increasing intensity to such an extent that it hampered her daily activities. On top of that she was also experiencing shortness of breath which was also progressive. However, she denied any orthopnea, paroxysmal nocturnal dyspnea (PND), fever, rash, altered bowel habits, cough, joint pain and any acute history of blood loss. According to the patient’s past medical history, she had on and off loose stools from 9 years of age, which resolved by age 16. Furthermore, 2 years back she was admitted to a nearby hospital with generalized weakness and jaundice. There is no official documentation but reportedly she was also transfused with 2 blood bags. Workup and diagnosis were not completed during her stay as she was non-compliant and left against medical advice at that time. All other tests were normal and her menstrual history was also normal. On examination, her vitals were blood pressure 110/60 mmHg, (reference, 120/80mm/hg); pulse 90 beats/minute (reference range, 70–100 beats/minute); temperature 98°F (reference range, 97–99°F) and respiratory rate 22 breaths/minute (reference range, 12–20 breaths/minute). Her general physical examination showed anemia, jaundice and clubbing, along with a raised jugular venous pulse. Her respiratory, cardiovascular system and central nervous examination were normal. However, her abdominal examination showed hepatomegaly (with liver palpable up to one finger) and splenomegaly (with spleen palpable up to 3 fingers below the costal margin) with the rest of the examination being normal.

Based on the history and examination, we ordered pertinent laboratory work up along with other tests. Her base line laboratory values were hemoglobin 2.3 g/dL (reference range, 11.1 – 14.5 g/dL); mean corpuscular volume (MCV) of 73.2 (reference range, 76 – 96); total lung capacity (TLC) 4.2×10 ^9^/L; platelets 92000/mm ^3 ^(reference range, 150 – 400000/mm ^3^); sodium 140 meq/L (reference range, 135–145 meq/L); potassium 3.6 meq/L (reference range, 3.5–5 meq/L); chloride 108 meq/L (reference range, 97–107 meq/L); calcium 8.5 meq/L (8.5–10.2 meq/L); magnesium 2.2 (reference range, 1.5–2.5 meq/L); total bilirubin 1.97 (reference range, 0.1–1.2 meq/L); serum glutamic pyruvic transaminase (SGPT) 59 units/L (reference range, 7–56 units/L); alkaline phosphatase (ALP) 123 IU/L (reference range, 44–147 IU/L); serum glutamic-oxaloacetic transaminase (SGOT) 40 units/L (reference range, 5–40 units/L); total protein 7.7 g/dL (reference range, 6–8.3 g/dL); albumin 3.5 g/dL (reference range, 3.5–5 g/dL) and prothrombin time to international normalized ratio (PT/INR) 11 seconds (reference range, 11–13.5 seconds).

As she presented with shortness of breath, we also performed an echocardiography and chest x-ray, both were within normal limits. At this point in time, we assumed that the patient’s symptoms and signs could be due to some autoimmune disorder (such as SLE, rheumatoid arthritis (RA)), immune mediated disorders (such as celiac disease), thyroid disorders (example immune thrombocytopenia purpura), Evan syndrome (due to thrombocytopenia and hemolytic anemia) and even chronic liver disease. Hence, in order to rule out the differentials and get to a possible diagnosis we conducted more tests. The patient iron profile showed ferritin 115.5 ng/mL (reference range, 12–150 ng/mL), serum iron 82 mcg/dL (reference range, 50–170 mcg/dL), total iron binding capacity (TIBC) 258 mcg/dL (reference range, 250–370 mcg/dL), transferrin saturation 37% (normal 25–35%) and her B12 levels were 2000 pg/mL (reference range, >200 pg/mL), both were in normal range. To rule out thyroid disorders, we checked her thyroid profile [thyroid stimulating hormone (TSH) 2.75 mU/L, (reference range, 0.5–4.0 mU/L); tri-iodothyronine (fT _3_) 2.45 pg/mL, (reference range, 2.3–4.2 pg/mL); thyroxine (fT _4_) 1.71 ng/dL, (reference range, 0.8–1.8 ng/dL)], which was within normal limits. She was also negative for anti-nuclear antibody, anti-Smith antibodies, anti-double stranded DNA antibody, anti-smooth muscle antibody and anti-mitochondrial antibody, thereby ruling out the possibility of autoimmune disorders. Furthermore, her blood Coombs test was also negative. To rule out secondary causes of idiopathic thrombocytopenia purpura (ITP), we tested for viral markers of human immunodeficiency virus (HIV), Epstein bar virus (EBV), cytomegalovirus (CMV) and performed a *Helicobacter pylori* test, all of which were negative. For celiac disease, we took anti-tissue transglutaminase (anti-TTG) titers of IgA and IgG, which came out to be 353 U/mL, (reference, <20 U/mL); and 419 U/mL (reference, <20 U/mL) respectively. The elevated titers confirmed the diagnosis of celiac disease. However, at this point in time, in order to improve symptoms of the patient, she was transfused with 2 bags of blood. Her bone marrow biopsy showed a positive coombs test with immune mediated hemolytic anemia and immune mediated thrombocytopenia. Hence, we established a diagnosis of celiac disease with Evans syndrome. However, in order to confirm our diagnosis of celiac disease, endoscopy was scheduled, but the patient did not consent to the procedure. Moreover, we started oral prednisone therapy 40 mg once daily which was tapered off and stopped in four weeks and the patient was counseled about a gluten free diet. Within these four weeks, the patient felt better and was discharged.

## Discussion

Celiac disease can result from gluten, several environmental triggers and immune factors. Gluten is the absolute protein component of wheat, whereas Gliadin is the alcohol-soluble fraction of gluten. The immune response to Gliadin leads to an inflammatory reaction in the small intestine, with infiltration of the lamina propria and epithelium with inflammatory cells along with villous atrophy. Furthermore, the development of celiac disease is highly linked to alleles that codes for HLA-DQ2 or HLA-DQ8 proteins, which are the products of the two HLA genes in adults. Women, due to unknown reasons, are more prone to the disease. Moreover, the frequency of autoimmune diseases in general is greater in women as compared to men and diseases like osteoporosis and iron deficiency anemia, which evoke work-up of celiac disease, are also common in women. The classic presentation is diarrhea, which may be associated with abdominal pain. However, studies have reported in the past decade that diarrhea is the chief complaint in less than 50% of cases. Moreover, with insufficient data on classic symptoms, the notion of silent celiac disease has arisen with the advent of serologic screening. Silent presentation includes iron deficiency anemia, incidental findings on endoscopy for other symptoms such as gastro esophageal reflux and osteoporosis. Furthermore, other less common findings can include constipation, neurologic symptoms, elevated liver enzymes, hypoproteinemia and hypocalcaemia. In addition, another study states that over the past 5 years, one third of the celiac disease patients were diagnosed by serological screening without gastrointestinal symptoms, indicating it can also be asymptomatic ^4, 5^.

Furthermore, hematologic manifestations of celiac disease consist of anemia (due to iron, folate or vitamin B12 deficiency), coagulopathy (due to vitamin K deficiency) and, very rarely, leukopenia and thrombocytopenia. Unlike in classic celiac disease, serum folate, vitamin B12 and ferritin can be normal in silent ^2^. Our patient had no folate, vitamin B12, iron or vitamin K deficiency, thereby further indicating the presence of silent celiac disease. In addition, the anemia present in our case was considered to be due to the autoimmune destruction of red blood cells. Our patient was female, and therefore at higher risk of celiac disease, and the only symptoms at presentation were generalized fatigue, shortness of breath and mild jaundice. The gold standard test for diagnosis of celiac disease is a duodenal biopsy, which shows the characteristic finding of intraepithelial lymphocytosis, crypt hyperplasia and villous atrophy. As far as the serological tests are concerned, sensitivity of both endomysial antibodies and anti-tissue transglutaminase antibodies (TTGA) is greater than 90%, with IgA tissue transglutaminase antibodies (TTGA) being more highly ranked ^4, 6^. In our case, we were unable to perform a confirmatory endoscopic biopsy as the patient did not consent to the procedure; therefore celiac disease was solely diagnosed from IgA tissue transglutaminase (TTGA) and IgG tissue transglutaminase (TTGA) levels.

Evans syndrome (ES), a rare condition affecting only 0.8% to 3.7% with either immune thrombocytopenia purpura (ITP) or autoimmune hemolytic anemia (AIHI), is diagnosed only after eliminating all other possibilities. Hence, other possible differentials for immune cytopenias such as systemic lupus erythematosus (SLE), acquired immunodeficiency syndrome and autoimmune lymphoproliferative disorders should be ruled out prior to diagnosis ^2, 3^. In our case, the patient had negative SLE workup and her HIV status was non-reactive. However, despite a negative coombs test from the blood sample, our patient later had a positive coombs test from a bone marrow sample indicating autoimmune hemolytic anemia (AIHA).

## Conclusion

Overall, the association of celiac disease with Evans syndrome (ES) is very rare. To our knowledge, only one study has reported 2 adult cases of patient having celiac disease and autoimmune hemolytic anemia (AIHA), with only one of the cases having both autoimmune hemolytic anemia (AIHA) and thrombocytopenia in the entire literature ^7^. Despite a rare coexistence of celiac disease and Evans disease (ED), we advise Coombs test (on both the blood and bone marrow sample) in all patients of celiac disease presenting with anemia with normal hematinics.

## Consent

Written informed consent for publication of their clinical details and clinical images was obtained from the patient

## Data availability

All data underlying the results are available as part of the article and no additional source data are required
