# Supplementary material for: Case Report: Rare comorbidity of celiac disease and Evans syndrome: The rare correlation of Celiac and Evans syndrome
Source: F1000Res. 2019 Feb 14;8:181. [Version 1] doi: 10.12688/f1000research.18182.1 (PMC6613476; doi:10.12688/f1000research.18182.1)
Supplement: Peer review history

# Peer review: Reviewer response for version 1

**DOI:** 10.5256/f1000research.19888.r50760

**Published:** 2019-07-05

**Authors:** Kofi Clarke

**Affiliations:** 1 Department of Medicine, Division of Gastroenterology and Hepatology, Penn State Health Milton S. Hershey Medical Center, Hershey, PA, USA

Case is written in great detail.

1. Evans syndrome is uncommon, and the association with celiac disease is extremely uncommon. Since the pretest probability is very low in patients with celiac disease, I would not recommend routine screening for Evans syndrome in all patients with celiac disease. It will be prudent to screen in only the appropriate clinical setting.
2. Recommend amend the first line in the discussion section celiac disease results from gluten ingestion in genetically predisposed individuals. There is no convincing evidence of disease onset only from environmental triggers.
3. It will be helpful to include a line in the history on excluding any medications/supplements that can cause hemolytic anemia.

I confirm that I have read this submission and believe that I have an appropriate level of expertise to confirm that it is of an acceptable scientific standard.

# Peer review: Reviewer response for version 1

**DOI:** 10.5256/f1000research.19888.r49207

**Published:** 2019-06-17

**Authors:** Lucia Terzuoli

**Affiliations:** 1 Department of Medical Biotechnologies, University of Siena, Siena, Italy

The case report is an interesting work, which adds another case to these rare comorbidities.

 The only point, which in my opinion, should be improved, is the bibliographical note no. 2 in the Introduction. The authors speak of "Several conditions such as determinatis herpetiformis, autoimmune thyroiditis and type 1 diabetes mellitus reported with CD, but in literature the bibliography refers to Evans syndrome. Perhaps it would be more correct to add other types of citations, such as, for example:

- Kotze LMDS *et al*. Immune mediated diseases in patients with celiac disease and their relatives: a comparative study of age and sex. Arq Gastroenterol 2018; 55:346-351 ^1^.
- Ferrari SM *et al*. The association of other autoimmune diseases in patients with Graves’ disease (with or without ophtalmopathy): review of the literature and report of a large series. Autoimmun Rev 2019; 18:287-292 ^2^.
- Nederstigt C *et al*. Associated auto-immune disese in type 1 diabetes patients: a systematic review and meta-analysis. Eur J Endocrinol 2019; 180:135-144 ^3^.

I confirm that I have read this submission and believe that I have an appropriate level of expertise to confirm that it is of an acceptable scientific standard.

## References

[1] : IMMUNE MEDIATED DISEASES IN PATIENTS WITH CELIAC DISEASE AND THEIR RELATIVES: A COMPARATIVE STUDY OF AGE AND SEX. *Arq Gastroenterol*.55(4) : 10.1590/S0004-2803.201800000-77 346-351 10.1590/S0004-2803.201800000-77 30785517

[2] : The association of other autoimmune diseases in patients with Graves' disease (with or without ophthalmopathy): Review of the literature and report of a large series. *Autoimmun Rev*.2019;18(3) : 10.1016/j.autrev.2018.10.001 287-292 10.1016/j.autrev.2018.10.001 30639646

[3] : Associated auto-immune disease in type 1 diabetes patients: a systematic review and meta-analysis. *Eur J Endocrinol*.2019;180(2) : 10.1530/EJE-18-0515 135-144 10.1530/EJE-18-0515 30508413

# Response

**Published:** 2019-06-18

**Authors:** Uzair Yaqoob

**Affiliations:** Dow University of Health Sciences, Pakistan

Thank you for your valuable comments
